# Research agenda setting with children with juvenile idiopathic arthritis: Lessons learned

**DOI:** 10.1111/cch.12904

**Published:** 2021-08-12

**Authors:** Karijn Aussems, Casper G. Schoemaker, Anouk Verwoerd, Wineke Ambrust, Katherine Cowan, Christine Dedding

**Affiliations:** ^1^ Department of Ethics, Law and Humanities Amsterdam UMC (Location VUmc) Amsterdam The Netherlands; ^2^ Department of Pediatric Rheumatology and Immunology, Wilhelmina Children's Hospital University Medical Center Utrecht Utrecht The Netherlands; ^3^ Department of Pediatric Rheumatology and Immunology, Beatrix Children's Hospital, University Medical Center (MCG) University of Groningen Groningen The Netherlands; ^4^ James Lind Alliance University of Southampton Southampton UK

**Keywords:** child participation, James Lind Alliance, juvenile idiopathic arthritis (JIA), patient involvement, research agenda setting

## Abstract

**Aim:**

The aim of this qualitative study is to understand the research priorities of Dutch children with juvenile idiopathic arthritis (JIA) as well as researching how children can be involved.

**Background:**

Several health research agendas have successfully been developed with adults but rarely with children. Children are still seldom recognized as possessing credible knowledge about their own body and life. This research project with focus group discussions and interviews with children with juvenile idiopathic arthritis (JIA) was an innovative addition to a nationwide prioritization of research questions of patients with JIA, their carers and health care professionals, based on the James Lind Alliance (JLA) methodology.

**Results:**

Children with JIA appreciated being invited to give their opinion on JIA research prioritization as knowledgeable actors. They have clear views on what topics need most attention. They want more insight on how to medically and socially treat JIA so that they can better fulfil their aspirations at school, later in work and with their relationships.

**Conclusion:**

We have identified the Top 5 research priorities for children with JIA. Most priorities are unique and differ from the priorities of the adolescents and young adults, parents and healthcare professionals in the main JLA priority setting exercise. Ultimately, two of the children's priorities were included in the final JLA Top 10.

**Key messages**

- The children's top research priority is the influence of JIA on future opportunities regarding school results, work and relationships.
- Adding qualitative research methods to the JLA approach enabled children to (collectively) reflect on their lives and to familiarize themselves with the role of agenda setting, research and the broad field it encompasses.
- Dialogue between children, young people, caregivers and healthcare professionals needs to be carefully facilitated to ensure all perspectives are mutually understood and acted upon.

## 1 INTRODUCTION

Patient and public involvement is increasingly recognized as an important factor in defining research priorities, to ensure research questions correlate with patients' burning questions and daily realities (Abma & Broerse, 2010; Odgers et al., 2018). Involving patients helps research to become more responsive to patient's needs, but also to do justice to the experiential knowledge of patients, as it complements medical and scientific knowledge, challenges what is conventionally known and reduces waste in research (Gillard et al., 2012; Macleod et al., 2014; Schölvinck et al., 2020). It is also underpinned by the democratic right to influence decisions affecting one's life (Thompson et al., 2014). So far, several research agendas have successfully been developed with adults but rarely with children (Hart et al., 2016). Unfortunately, children are still seldom recognized as possessing credible knowledge about their own body and life. The lack of agenda setting with children is probably also triggered by the limited insight and expertise in *how* best to include their voice (Bate et al., 2016; Bird et al., 2013; McDonagh & Bateman, 2011; Thompson et al., 2014) and the idea that medical professionals and adults know best (Gibbs et al., 2018).

Social science research has shown that even young children are willing and able to be involved in research (Alderson, 1992; Christensen & Prout, 2002; Dedding, 2009; James & Prout, 1997; Nap‐van der Vlist et al., 2019) and that they offer a unique perspective (Lems et al., 2020; Sarti et al., 2017; Schalkers et al., 2014; van Bijleveld et al., 2020). Together with the promotion of the international rights of children for their opinion to be taken into account (UN General Assembly, 1989), this has led to their stronger direct engagement in research (instead of their guardians representing them) and to a shift from researching on children to researching with children (Bird et al., 2013). Therefore, the question is not whether children could be involved in research but *how* to do this in a meaningful way. That is, working together with respect for their needs and competencies, and their voice to be heard and acted upon (Dedding et al., 2013; Gibbs et al., 2018).

This project with children with juvenile idiopathic arthritis (JIA) was part of a nationwide prioritization of research questions of patients with JIA, their carers and healthcare professionals, based on the James Lind Alliance (JLA) methodology. The JLA was founded in 2004 to facilitate Priority Setting Partnerships (PSPs) with patients, carers and clinicians. Their methodological approach is used worldwide, albeit rarely with children. We found one JLA PSP that worked with children only, namely, children requiring elective surgery for conditions affecting the lower limbs (Vella‐Baldacchino et al., 2019) and one in progress, concerning children with cancer.^1^ Further, one research project on research priority setting in the United States was conducted with children with rheumatic diseases and their carers living in the United States. However, children from the ages below 13 years were excluded, and only caregivers attended focus groups (Correll et al., 2020).

The research protocol (Schoemaker et al., 2018) and the outcome for the nationwide research patient priorities have been described elsewhere (Verwoerd et al., 2021); a process evaluation of the whole project is also published separately (Jongsma et al., 2020). This article solely focuses on the outcomes and the qualitative research methods that were additional to the standard JLA approach. The aim of our study was to explore the research priorities of young children with JIA, and to reflect on the process and methodologies used in order to guide future JIA research and funding to the issues that matter most to them. Furthermore, we aim to improve our understanding of how to meaningfully involve children in agenda‐setting processes.

## 2 PATIENTS AND METHODS

### 2.1 Generating questions

**FIGURE 1:**
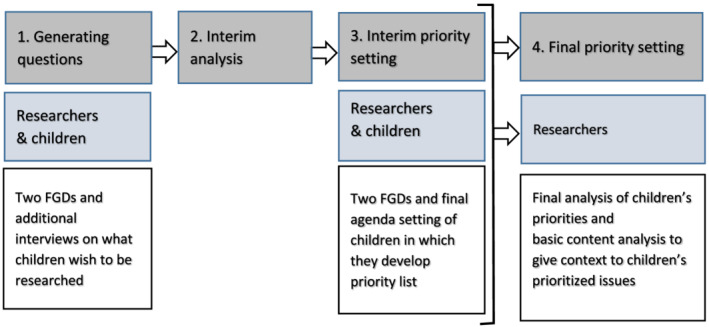
A flow chart of the participation of children, in addition to the model of Verwoerd et al. (2021, fig. 1) showing the regular James Lind Alliance (JLA) process in grey and in blue and white the additional research activities. FGDs, focus group discussions

This qualitative study used focus group discussions (FGDs) of 1 h each and interviews of approximately half an hour to involve children, aged 9–13, with a diagnosis of JIA, to formulate and prioritize research questions. We recruited our participants via convenience sampling. The Dutch Juvenile Arthritis Association and paediatric rheumatologists of two academic centres shared our invitation with their members/patients to participate in this research. CD carried out two FGDs at the annual patient information day, organized by the Dutch Juvenile Arthritis Association (November 2018). Further, a research assistant conducted additional interviews at two academic centres (March 2019). The aim of these interviews was to involve more and younger children, and to ensure engagement of non‐members of the patient organization. Additionally, as part of the regular JLA approach, children could share their ideas in an online and hardcopy survey (December 2018–March 2019) (Figure 1).

A cartoon was used to communicate the aims of the project. We explained that researchers need to know what it means to live with JIA, what challenges children experience and what research they think needs to be done to improve their (everyday) lives, in order to do research that is relevant to them.

During both the FGDs and interviews, we started with a warm‐up activity to help the children to familiarize themselves with the researchers and with each other and to feel comfortable and included. To ensure that the children were not alienated from their own experiences and lives in the process, we started by using images that represented different domains of their lives.

### 2.2 Analysing questions

First, we analysed the transcripts of the first two FGDs and the interviews using Microsoft Word comments and reached agreement on the questions that the children wanted to be studied, and delivered the list of questions to the PSP steering group. The steering group received questions from patients of *all* age ranges, carers and healthcare professionals and categorized these into summary questions (Verwoerd et al., 2021). The summary questions based on questions of children were categorized into main themes for the children and used as a starting point for the discussion about their research priorities.

### 2.3 Interim session

**FIGURE 2:**
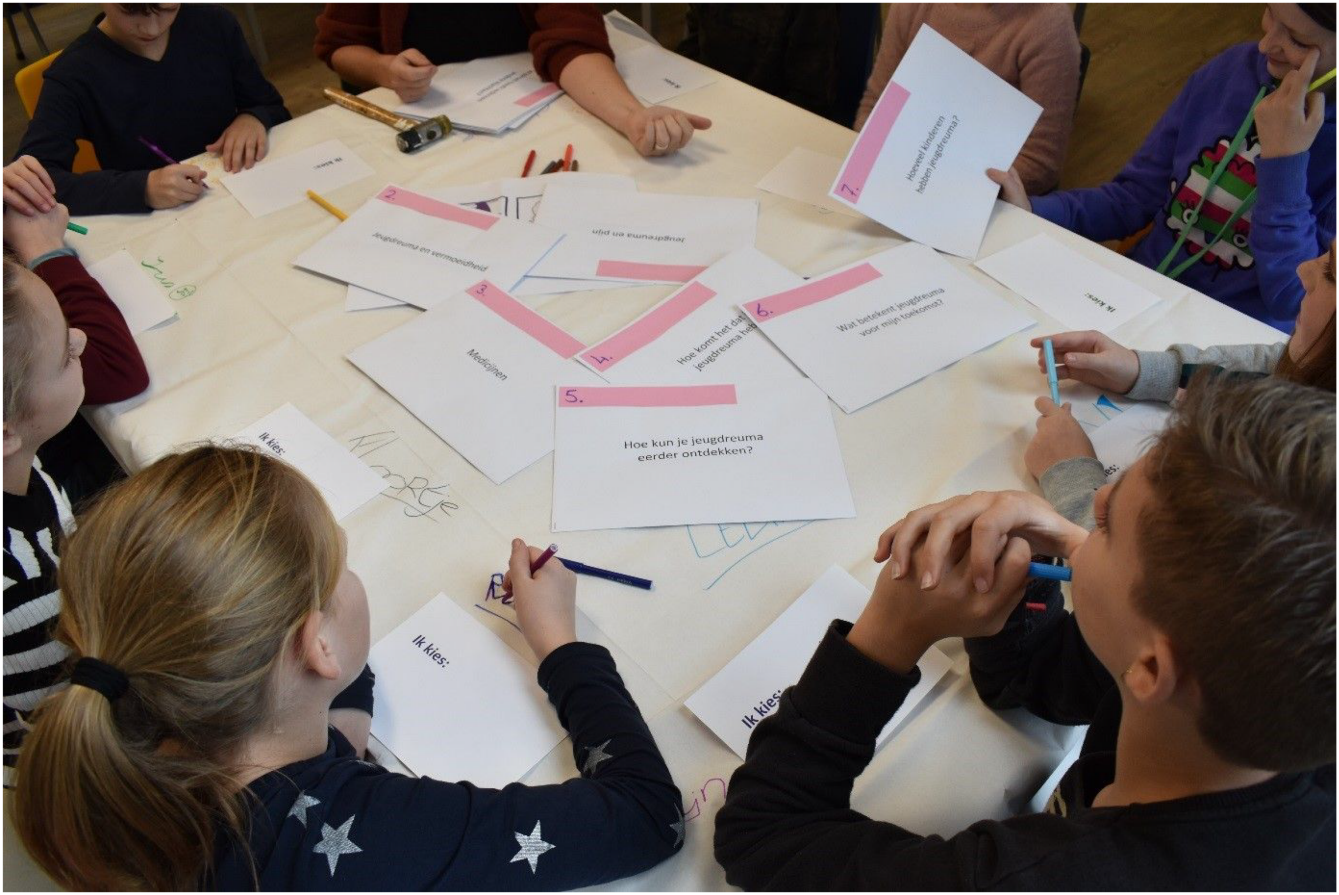
Children during the interim agenda setting. They view the envelopes with the main themes and write down their personal preference

The next step involved organizing two sessions for the children's interim agenda setting (CD and KA), again at the annual patient information day (November 2019). The main themes were written on envelopes and presented in two separate meetings to the children. First, each child shared in the group what their personal preferred theme was and why (see Figure 2). The children were then able to open envelopes that contained the corresponding summary questions to help them to narrow down their preferred research questions. We then invited them to jointly discuss and decide the Top 5 themes of the whole group, leading to a final Top 5 of research questions for each group. Next, the two groups compared their respective Top 5 and then, through facilitated discussion, formulated a final list in order of preference. Briefly after the FGDs, the children physically handed this final list in a golden envelope to the PSP steering group. Afterwards, we analysed the chosen themes and how they supported their choice so that we could select the best corresponding summary questions (see Table 3).

### 2.4 Final analysis

The list of the children's research priorities, and their arguments as articulated during the process, was used for a basic content analysis (Hsieh & Shannon, 2005) (KA and CD), in order to give context for their prioritized issues.

## 3 RESULTS

In total, 40 children (24 girls and 16 boys, 9–16 years, living with JIA for between 4 months and 9 years) participated in the FGDs and interviews (see Table 1). Further, 42 children of the same age bracket filled in the surveys. On the first online survey of this PSP, no more than 23% of responding patients were younger than 16 years, and only 16% attended primary school. On the second survey, no more than 2.4% of the responding patients attended primary school (Verwoerd et al., 2021). By undertaking the phases of the JLA PSP, saturation was reached on the identified research questions of the children.

**TABLE 1:** Characteristics of focus group participants (*n* = 31) and interview (*n* = 9)

Phase	Session	Date	No respondents (*N* = 40)	Age	Gender
Generating questions	FGD 1	November 2018	7	12–14	5 girls 2 boys
	FGD 2	November 2018	8	10–14	5 girls 3 boys
	Group interview	February 2019	3	11–15	2 girls 1 boy
	Duo interview	February 2019	2	9–10	2 girls
	Interviews (of which 1 phone call)	March 2019	4	15–16	3 girls 1 boy
Interim survey	FGD: Working Session 1	November 2019	9	10–12	5 girls 4 boys
	FGD: Working Session 2	November 2019	7	10–15	4 girls 3 boys
	FGD: final priority setting		11^a^	10–15	6 girls 5 boys

Abbreviation: FGD, focus group discussion.

^a^ Out of the 16 children who participated in the interim priority setting, 11 took part in the children's final priority setting stage.

In Part A, we first describe the Top 5 research priorities identified by the children, followed by an explanation of why this is of importance to them. In Part B, we reflect on the process and lessons learned.

### 3.1 Part A: Children's priorities for research

The first two FGDs and the interviews provided 81 questions. These questions were added to the PSP question database of the survey as part of the JLA methodology. The steering group received in total 604 questions of patients, carers and healthcare professionals and categorized these into 53 summary questions (Verwoerd et al., 2021). Twenty‐three of these 53 summary questions included the questions that were upraised by the children. Furthermore, the children who had participated in the online or hardcopy survey also delivered additional unique research questions, which were categorized under mostly the same summary questions. Five extra summary questions were added.

We felt we needed to focus the discussion. Therefore, we omitted the 25 summary questions that were not based on original questions from children. As a result, the children did not have to discuss ‘uninteresting’ summary questions. This led to a total of 28 summary questions based on concerns raised directly by children. Further, we thought 28 questions were too many to discuss. Therefore, we grouped them into 12 themes (see Table 2) and reworded them into child‐friendly language so that children could easier oversee it and make priorities.

**TABLE 2:** Twelve themes with 28 corresponding questions from the 53 summary questions as formulated by steering group

No.	Theme	Question
1	JIA and pain	a. How can pain best be recognized and be treated (with medication), and what action can a patient take him/herself? b. Pain is often present when the disease is in remission. How does this happen, what can one do about it, and can one predict which patients will suffer from them?
2	JIA and fatigue	a. Why are children with JIA fatigued more quickly, what can be done about it, and how can one best cope with the fatigue in daily life? b. Why do children with JIA have sleeping problems and what can be done about it? c. Fatigue is often present when the disease is in remission. How does this happen, what can one do about it, and can one predict which patient will suffer from them?
3	Medication	a. Administering of medication (injections, tablets) ‐ How can pills be manufactured in such a way that they are easy to take? (i.e. shape, color, taste) ‐ How can the injections be less painful? b. Side effects of medication ‐ How do the side effects of Methotrexate (MTX) develop (e.g. nausea) and can you predict who will (not) get it? ‐ How can the side effects of Methotrexate (MTX) be prevented? ‐ What are the short‐ and long‐term side effects/consequences of the drugs taken for JIA? c. Tapering off medication ‐ When and how can medication for JIA best be tapered off? d. Alternative treatment ‐ Are there any strategies in alternative medicine that can help alleviate health complaints of JIA?
4	Causes of JIA	a. How does JIA develop, and which factors influence this? b. Is JIA inheritable, and if yes, in what way?
5	How can JIA sooner be diagnosed?	a. How can we better and sooner recognize JIA? b. Can JIA be prevented, and if yes, how?
6	JIA and future	a. How many patients with JIA outgrow it? b. What is the influence of JIA on future opportunities regarding school results, work and relationships? c. How can JIA be cured?
7	Prevalence	a. How many people in the Netherlands have JIA?
8	Why do people have different symptoms?	a. How can the course (flares, extensions, cure) of JIA be better explained and predicted? b. What is the reason people with JIA do not all have the same symptoms (for example problems with eyes and joints)?
9	Misconceptions in my environment	a. How do you prevent misconceptions in the immediate environment of children with JIA?
10	Dealing with emotions	a. How best can I deal with my feelings regarding JIA and communicate about this with others?
11	Care that meets my needs	a. How can the care and guidance of patients with JIA best be adjusted to the needs of the children? b. What can you do in person to manage your JIA well?
12	JIA and sports	c. How do you need to deal with pain during sports? d. What is the best way to practice your favorite sport safely?

Abbreviation: JIA, juvenile idiopathic arthritis.

Table 3 shows the results of the interim priority setting sessions where children with JIA chose their final Top 5, followed by an explanation of why these matter to them.

**TABLE 3:** Summary questions as prioritized by the children during their final priority setting (*n* = 11)

Interim Top 5 of the children of Group A^a^	Interim Top 5 of the children of Group B	Final Top 5 of the children of Groups A and B
1. What is the influence of JIA on future opportunities regarding school results, work and relationships?	1. What is the influence of JIA on future opportunities regarding school results, work and relationships?	1. What is the influence of JIA on future opportunities regarding school results, work and relationships?
1. Why are children with JIA fatigued more quickly, what can be done about it, and how can one cope with the fatigue in daily life?	2. How can pills be manufactured in such a way that they are easy to take? (i.e. shape, color, taste)	2. Why are children with JIA fatigued more quickly, what can be done about it, and how can one cope with the fatigue in daily life?
2. What is the best way to practice your favorite sport safely?	3. How can pain best be recognized and be treated (with medication), and what action can a patient take him/herself?	3. How can pain best be recognized and be treated (with medication), and what action can a patient take him/herself?
3. How can pain best be recognized and be treated (with medication), and what action can a patient take him/herself?	4. Causes of JIA: How does JIA develop, and which factors influence this and (b) is JIA inheritable, and if yes, in what way?	4. How can pills be manufactured in such a way that they are easy to take? (i.e. shape, color, taste)
4. Pain is often present when the disease is in remission. How does this happen, what can one do about it, and can one predict which patients will suffer from them?	5. Why are children with JIA fatigued more quickly, what can be done about it, and how can one cope with the fatigue in daily life?	5. What is the best way to practice your favorite sport safely?

Abbreviation: JIA, juvenile idiopathic arthritis.

^a^ The children chose to put two themes in joint first place.

[Corrections made on 5 September 2021, after first online publication: In Table 3, the numbering in the first column has been updated in this version.]

#### 3.1.1 Impact on (future) school results, work and relationships

The children stressed the impact of JIA on school results, work and relationships. First, almost all the children experienced a strong impact from JIA on their schooling, as it hindered their mobility at school:

At secondary school you have three to five stairs. …. In the end of the day you feel your knee. [I don't use the lift] because then I can't be in class in time, because it takes long before the lift arrives. (Thijs, 15)

Ruba (15) wanted for research about mobility at school: ‘Could they invent a way that enables me to easier open the lockers at school?’ Chantal noted the limitations to her progress at school, ‘we have a very important … day with exams … but that day I have to be at the intravenous drip.’ Erik (15) proposed research to help find out ‘a way that you can still join the class while you're actually not there’.

Second, the children talked about their future employment and their concerns due to their JIA. Sasha (15) confided: ‘I wanted to become a professional footballer, or work in a restaurant, but I can't do that.’ Others still saw options while being cautious: ‘I know what I want to become, a child psychologist and I hope I can do that. Maybe I can't do that because maybe school is too heavy’ and ‘I actually want to work for the police, definitely won't work. Maybe I can become a doctor, I don't know if that's possible.’ On the other hand, some primary school children found it hard to think about their future, like Eva (10): ‘I'm still too young to think about the future, I'll see what will happen by then.’

Third, they often mentioned how their parents, classmates and bystanders are either of help or hindrance in meeting their needs. Freek (12) felt hindered: ‘I'm not allowed to try out many things because my parents don't want that. They also don't want me to do the gymnasium.’

Eline (16) appreciated her friend's care, ‘I have a friend, she always asks how I'm doing and what I've done. … I've always appreciated it.’ However, she also encountered disapproval from others: ‘I've been told that I'm an attention seeker. They didn't understand that I suffered a lot and that I was sick very often.’ This was mentioned by three other teens, including Janet (12), who was called lazy: ‘If you're firstly in your wheelchair and then you get out, people look strangely at me, think it is fake. …. Sometimes people say that I'm lazy.’ Tessa (15) tried to avoid criticism, which created a distance between her and her friends:

I'm always troubled that I have to go to hospital. Then I'm more quiet at school, cannot really focus, find it very difficult to speak about it with friends. I do not want to appear like I want attention.

#### 3.1.2 Causes of fatigue and how to cope with it

The second question the children prioritized was why they are fatigued more quickly, what can be done about it and how one can best cope with fatigue in daily life. Several children thought this was the most important question, like Bas (10): ‘Because I'm very often tired, many things I can't do, like music and play.’ Jordy (11) sighed: ‘I have … problems with fatigue … because I almost never sleep.’ Merel proposed the question, ‘why are you tired at school? I can't concentrate well because I'm tired.’ They questioned the reasons for often being tired (‘how that is possible’) and what could be done about it (‘wanting that there is something against it’).

#### 3.1.3 Recognizing and treating pain

The third priority question was how pain can be best recognized and treated (with medication), and what they can do themselves, as a patient. First, they spoke of pain that hinders them in their actions:

If you fall at a party, then your knee or ankle gets swollen, and then you need to go home. It's a pity, because some, if they fall, they have less pain and then it's not such a problem and they can continue after five minutes. (Jora, 12)

Another child blamed the pills for his pain: ‘If the pills don't work [when I'm at school] then I get pain in my fingers or heel.’ Bo (11) requested research that will lead to a pill that quickly removes the pain ‘because sometimes it's really inconvenient’. Pascal wanted to know why pills only work temporarily: ‘I've experienced pills that work during inflammations, but later on the pain returns. I want to understand why.’

Second, the children also encountered moments that the pain starts afterwards, like Kim (11) who even felt it only a day later: ‘At the party you enjoy, and you don't feel anything … and the next day [the JIA] begins to bother you.’ They proposed the research question ‘why don't we feel pain when we are busy, but thereafter we do?’ Third, besides research on pain reduction, they wanted research on how they can cope with ongoing pain. Jenny (16) emphasized: ‘People shouldn't laugh away the pain’, meaning that other people need to take their problems with pain seriously.

#### 3.1.4 Better administering of medication

The fourth question that the children prioritized concerned the administering of medication, namely, how the injections can be less painful, if there is an alternative medicine in the shape of a pill as a replacement for the needles and infusions, and how pills can be made that are easy to take (shape, colour and taste).

Bo (11) disliked going to the hospital for a whole day per month to get an IV drip: ‘I want to know if everything that I get can be put in a pill because otherwise I have to miss the whole school day and that isn't so nice.’ Children also suggested that research should investigate how pain when taking medication can be avoided: ‘I find these injections horrible’ and ‘imagine if they turn it into pills, then you don't need these injections anymore.’ Tessa (16) expressed a similar wish, based on her experience:

Every two weeks I get an injection. Before I used to get pills. They made me nauseous for a whole weekend. The injection only bothers me when they inject. Although it is better, it would even be better if they can give it to me like pills. …. Now I have to wait for nurses who inject me at home. A pill is easier, and I just do not like injections. If I go to the hospital, I dislike the blood tests most.

Besides nausea as a side effect of the pills, the children also criticized their smell, taste and shape: ‘The pills are so big’, ‘in the beginning I had pills with a very dirty smell, that it almost made you to vomit’, ‘sometimes [the pills] dissolve in the mouth before you could swallow them’ and some have a taste ‘like puke, poo and diarrhea’.

#### 3.1.5 Safe practice of sports

The fifth question that the children prioritized was about sports: ‘What is the best way to practice your favorite sports safely?’ Eva (10) described being comforted by friends if she is tired during sports: they tell her to stand on the side for a moment, and they sometimes join her, ‘I think that's sweet.’ Bas (10) gave up on sports: ‘I've quit playing soccer. …. Every week crying on the sideline, because I had so much pain.’ Thijs (15) missed the socialization since he quit:

I usually had soccer in the evening, but not anymore. I did it since age seven. But I cannot do it anymore with my knee. That's not so nice. [I miss] the fun with soccer and friends and all, because you almost do not meet them anymore.

Some children wondered why their doctor or parents do not allow them to exercise, as it was not explained to them. They suggested that research could find out how they can be supported to exercise, for example, research on pills to reduce the pain or equipment that improves their mobility. Erik (15) wanted ‘to find a permanent way to at least stay more physically active or that your knee stays thin. Not really that the rheumatic disease disappears, but that you at least don't have swollen knees or wrists’. Berend (10) proposed the invention of equipment: ‘a thing to sit on, but one that moves fast, easy to steer, one that you don't only sit on and that doesn't go slow.’

Erik (13) shared his coping strategy for managing the pain and fatigue afterwards: ‘If I play [computer] games too long, I get pain in my fingers (…) [then] I lay on my bed or do something else.’

### 3.2 Part B: Reflections on the methodology

Most of the children were pleasantly surprised that they were asked to think about what should be researched for children with JIA. It made them feel important and recognized as serious stakeholders. Although some spontaneously shared some first thoughts, most needed time to get acquainted with the purpose of the consultation. Creating an informal and relaxed atmosphere, starting from their own experiences and lifeworld, the supporting tools (the cartoon and the template) and the interaction with the experienced facilitators and their peers, helped them to get started.

We know from our work in the field of participatory health research that participation is most meaningful if it starts from the lifeworld of participants and the process is carefully facilitated, with room for flexibility (Dedding et al., 2013). Therefore, we did not simply *collect* questions but jointly *generated* questions, jointly because this enabled children to develop their voice in a safe space of their own peer group. Through dialogue, they could reflect on their (common) issues and impact of JIA on their lives, and what matters most to them. Working together with peers, feeling understood, mobilized their energy and sense of empowerment.

Although we originally aimed to work with children aged 10–13, in practice, we broadened the criteria. This was partly because the patient organization did not want to exclude enthusiastic members but also because it touches upon key principles of participatory health research, namely, shared ownership, being locally situated and inclusion (the right to have a say) (Abma et al., 2019; Dedding et al., 2013; International Collaboration for Participatory Health Research [ICPHR], 2013).

Some children found it hard to let go of their personal priority list, which was at times different from other children's priorities during the same session. It therefore needed sensitive guidance, despite the limited time and the children's impatience, to come to an agreement. The fact that we had chosen for a Top 5, instead of the JLA's usual Top 10, turned out to be successful. A Top 10 would have required more discussion time. Because the concentration of the children was limited, an extra session would have been required, and arranging this at a time that suited all the children would have been challenging, particularly on short notice. An extra session, however, would be an opportunity to incorporate greater diversity, such as age, disease and symptoms, which was not possible in the current process.

These focus groups with young children clearly added to the standard JLA methodology, which is mostly based on online input. Consequently, younger children are often under‐represented. This underscores the significance of adding focus groups to JLA PSPs about paediatric diseases. Without these focus groups, the views of adolescent and young adult patients will be over‐represented.

Furthermore, working together with children within the nationwide PSP facilitated knowledge sharing and exposure but was done in parallel rather than integrated into the discussions with the other stakeholders. In this project, the priorities of children were discussed in the final overall priority setting meeting, with the presence of young adults who promoted the choices of the children in their absence. Finally, two out of five of the children's research priorities ended up in the final joint Top 10 for all stakeholder groups (see Table 4).

**TABLE 4:** Final ranking of the Top 20 research questions and the rankings per group in the interim priority setting (Verwoerd et al., 2021)

No.	Question	Ranking patients in the main JLA trajectory	Ranking carers in the main JLA trajectory	Ranking clinicians in the main JLA trajectory	Ranking children in additional qualitative trajectory
1	Pain and fatigue are often present when the disease is in remission. How does this happen, what can one do about it, and can one predict which patients will suffer from them?	3	10	1^a^	
2	What is the best treatment plan for each individual patient? (e.g. start a biological directly, which one, what to do when the first one does not work, and how can medication best be tapered off?)	42	28	7	
3	What is the best treatment plan for uveitis in JIA, and are there factors that predict its effectiveness?	36	25	5	
4	Why are children with JIA fatigued more quickly, what can be done about it, and how can one best cope with the fatigue in daily life?	6^a^	6	1^a^	2
5	How does JIA develop and which factors influence this?	6^a^	5	24	
6	How can the course (flares, extensions, cure) of JIA be better explained and predicted?	15	9	6	
7	What is the influence of nutrition on JIA, and can a diet help?	2	2	7	
8	What are the short‐ and long‐term side effects/consequences of the drugs taken for JIA?	8	1	10	
9	What is the influence of JIA on future opportunities regarding school results, work and relationships?	9	11	20	1
10	What is the influence of sports and exercise on JIA and vice versa?	24	37	7	
The following questions were also discussed and put in order of priority at the workshops:					
11	What are the long term physical consequences of JIA?	1	3	10	
12	How can JIA be cured?	4	4	42^a^	
13	Is there an association between JIA and other (autoimmune) diseases, and if yes, how can one better understand this?	10	8	42^a^	
14	How can pain best be recognized and be treated (with medication), and what action can a patient take him/herself?	32	30	29^a^	3
15	Which knowledge and skills are needed for patients and parents to achieve a healthy and active lifestyle?	38	24	4	
16	How can pills be manufactured in such a way that they are easy to take? (e.g. shape, color, taste)	29	40	29^a^	4
17	How can children/adolescents with JIA best be supervised?	10	15	10	
18	IS JIA inheritable, and if yes, in what way?	5	13	51	
19	What is the best way to practice your favorite sport safely?	43	49	17	5
20	Are there any strategies in alternative medicine that can help alleviate health complaints of JIA?	21	7	42^a^	

^a^ Signifies that a question is ranked in joint place with another question.

## 4 DISCUSSION

The objective of this project was to engage children in a national JIA research agenda‐setting process to improve the relevance of JIA research. In this additional qualitative trajectory, 40 children with JIA shared their personal experiences and what they think is the most important to study, namely, the impact of JIA on their (future) opportunities regarding school results, work and relationships. They look at their JIA not only as an inhabitant of their body but also as a person who wants to belong and take part in normal everyday life. This may not come as a surprise, as young people with JIA in earlier studies showed that JIA impairs children's capacity for social participation (Tong et al., 2012) and triggers fears for the future, feeling unsure of the physical, psychological and social impact of JIA in their lives (Eyckmans et al., 2010) including being rejected if they tell others about their JIA (van Gulik et al., 2020).

Three questions in the children's Top 5—numbered 14, 16 and 19 in Table 4—were ranked much lower by the other three groups. This illustrates the added value of the focus groups (Verwoerd et al., 2021). Two out of five of their research priorities ended up in the final joint Top 10. In the PSP's final priority setting workshop, a group of five young adult patients, five parents of patients and ten healthcare professionals paid attention to the children's unique perspective and acknowledged the importance of engaging children as important stakeholders in JIA care. They however had the huge challenge to do justice to *all* perspectives (patients, parents and professionals) and *all* age groups. A main difference with the final Top 10 priorities determined by the PSP process is that the children in this research prioritized questions on their *belonging*: how to actively live their lives with their family, classmates, friends and sports mates like anyone else. For this, they wanted *both* psychosocial and medical questions to be answered. This resembles the views of young people with JIA in the United Kingdom in earlier research on their preferred research agenda (Parsons et al., 2017). They had been critical of the fact that psychosocial topics are not sufficiently researched. However, their top research priority was on the best ways of providing support to recently diagnosed young people. They felt that, in this phase, little support is available to them. In another research project identifying research priorities among children with rheumatic disease and their carers in the United States, the outcome was that ‘finding new treatments’ was their priority (Correll et al., 2020). The authors mention that the differences between the results in the United States and the United Kingdom could be related to disease‐ and culture‐specific outcomes. However, this may also be related to differences in age and lived experience between the children and parents, as is the case between the children and parents in the Netherlands (Verwoerd et al., 2021). It may also be due to differences between the research approaches, as children attended FGDs in both the United Kingdom and the Netherlands, unlike in the United States where children only participated in the online surveys, so the questions of the survey and parents may have influenced the children's answers.

Clearly, this research shows the importance of listening to children as they have a unique perspective. Moreover, children appreciated being acknowledged as knowledgeable and capable conversation partners. This calls for a strong integration of children's research priorities in future research proposals and financial support for implementation.

## 5 STRENGTHS AND LIMITATIONS

Most of the involved children were members of the patient society. Working closely together with a patient organization provided the opportunity to bring children from various parts of the country together at the annual event, although it also created a selection bias. We therefore deliberately reached out to interview children in hospitals. However, these individual children were not part of the final working sessions, as these took place at one of the annual events. By undertaking the phases of the JLA PSP, saturation was reached on the identified research questions of the children. However, future research could explore these research questions in more depth.

The difference between the agenda of the children and the final JIA PSP Top 10 research priorities cannot solely be attributed to their age. The difference in approach and methods might also be a reason. The advantages of interviews and FGDs were multiple. First, parents were not involved in the discussion, whereas for the survey, they were likely to be supervising the children's participation. Second, through the discussions, the children had the opportunity to learn more about the broad field of research. Third, in the FGDs, they could reflect together with peers, which strengthened their confidence in articulating their priorities. Fourth, both methods were deliberately designed to start from their *lifeworld*—*ensuring genuine participation* to share their experiences. Finally, carefully listening to them provides context for their prioritized topics. These reflections might also be relevant for guiding agenda‐setting processes with adults next to the current approach.

## CONFLICT OF INTERESTS

The authors have no potential conflicts of interest to declare.

## ETHICS STATEMENT

The Medical Research Ethics Committee of University Medical Center Utrecht confirmed that this study was exempted from the Medical Research Involving Human Subjects Act (WMO) (METC Protocol Number 18‐721/C). Verbal consent of both the children and their parents was obtained. They were informed that they had the chance to withdraw this consent at any time of the research without giving a reason, that they were not obliged to answer questions and that their stories would be handled anonymously (by using pseudonyms).

## Data Availability

The raw data of this research project cannot be shared due to ethical reasons.

## ENDNOTE

^1^
Children's Cancer and Leukaemia Group. Children's Cancer Priority Setting Partnership. https://www.cclg.org.uk/Childrens-Cancer-PSP.

## References

[cch12904-bib-0001] Abma, T. , Banks, S. , Cook, T. , Dias, S. , Madsen, W. , Springett, J. , & Wright, M. T. (2019). Participatory research for health and social well‐being. Cham: Springer International Publishing. 10.1007/978-3-319-93191-3

[cch12904-bib-0002] Abma, T. A. , & Broerse, J. E. (2010). Patient participation as dialogue: Setting research agendas. Health Expectations, 13(2), 160–173. 10.1111/j.1369-7625.2009.00549.x 20536537 PMC5060528

[cch12904-bib-0003] Alderson, P. (1992). Everyday and medical life choices: Decision‐making among 8‐ to 15‐year‐old school students. Child: Care, Health and Development, 18(2), 81–95. 1587012 10.1111/j.1365-2214.1992.tb00343.x

[cch12904-bib-0004] Bate, J. , Ranasinghe, N. , Ling, R. , Preston, J. , Nightingale, R. , & Denegri, S. (2016). Public and patient involvement in paediatric research. Archives of Disease in Childhood – Education and Practice, 101(3), 158–161. 10.1136/archdischild-2015-309500 26802108

[cch12904-bib-0005] Bird, D. , Culley, L. , & Lakhanpaul, M. (2013). Why collaborate with children in health research: An analysis of the risks and benefits of collaboration with children. Archives of Disease in Childhood – Education and Practice, 98(2), 42–48. 10.1136/archdischild-2012-303470 23303525

[cch12904-bib-0006] Christensen, P. , & Prout, A. (2002). Working with ethical symmetry in social research with children. Childhood, 9(4), 477–497. 10.1177/0907568202009004007

[cch12904-bib-0007] Correll, C. K. , Dave, M. , Paul, A. F. , Gaizo, V. D. , Schrandt, S. , Partovi, R. S. , & Morgan, E. M. (2020). Identifying research priorities among patients and families of children with rheumatic diseases living in the United States. The Journal of Rheumatology, 47(12), 1800–1806. 10.3899/jrheum.190934 32062607

[cch12904-bib-0008] Dedding, C. (2009). Delen in macht en onmacht. In De Mondigheid Van Kinderen Uit Zich Vaak in Stilte. Amsterdam: Universiteit van Amsterdam. PhD thesis.

[cch12904-bib-0009] Dedding, C. , Jurrius, K. , Moonen, X. , & Rutjes, L. (2013). Kinderen en jongeren actief in wetenschappelijk onderzoek: Ethiek, methoden en resultaten van onderzoek met en door jeugd. Houten: Lannoo Campus.

[cch12904-bib-0010] Eyckmans, L. , Hilderson, D. , Westhovens, R. , Wouters, C. , & Moons, P. (2010). What does it mean to grow up with juvenile idiopathic arthritis? A qualitative study on the perspectives of patients. Clinical Rheumatology, 30(4), 459–465. 10.1007/s10067-010-1444-0 20383546

[cch12904-bib-0011] Gibbs, L. , Marinkovic, K. , Black, A. L. , Gladstone, B. , Dedding, C. , Dadich, A. , O'Higgins, S. , Abma, T. , Casley, M. , Cartmel, J. , & Acharya, L. (2018). Kids in action: Participatory Health Research with Children. In M. Wright & K. Kongats (Eds.), Participatory Health Research. Cham: Springer. 10.1007/978-3-319-92177-8_7

[cch12904-bib-0012] Gillard, S. , Simons, L. , Turner, K. , Lucock, M. , & Edwards, C. (2012). Patient and public involvement in the coproduction of knowledge. Qualitative Health Research, 22(8), 1126–1137. 10.1177/1049732312448541 22673090

[cch12904-bib-0013] Hart, R. I. , Mcdonagh, J. E. , Thompson, B. , Foster, H. E. , Kay, L. , Myers, A. , & Rapley, T. (2016). Being as normal as possible: How young people ages 16–25 years evaluate the risks and benefits of treatment for inflammatory arthritis. Arthritis Care & Research, 68(9), 1288–1294. 10.1002/acr.22832 27040737 PMC5042182

[cch12904-bib-0014] Hsieh, H. F. , & Shannon, S. E. (2005). Three approaches to qualitative content analysis. Qualitative Health Research, 15, 1277–1288. 10.1177/1049732305276687 16204405

[cch12904-bib-0015] International Collaboration for Participatory Health Research . (2013). Position paper 1: What is participatory health research? Version: Berlin: International Collaboration for Participatory Health Research.

[cch12904-bib-0016] James, A. , & Prout, A. (1997). Constructing and reconstructing childhood contemporary issues in the sociological study of childhood. London: Psychology Press.

[cch12904-bib-0017] Jongsma, K. , van Seventer, J. , Verwoerd, A. , & van Rensen, A. (2020). Recommendations from a James Lind Alliance priority setting partnership—A qualitative interview study. Research Involvement and Engagement, 6(1), 68. 10.1186/s40900-020-00240-3 33292829 PMC7678261

[cch12904-bib-0018] Lems, E. , Hilverda, F. , Sarti, A. , van der Voort, L. , Kegel, A. , Pittens, C. , Broerse, J. , & Dedding, C. (2020). ‘McDonald's is good for my social life’. Developing health promotion together with adolescent girls from disadvantaged neighbourhoods in Amsterdam. Children & Society, 34(3), 204–219. 10.1111/chso.12368

[cch12904-bib-0019] Macleod, M. R. , Michie, S. , Roberts, I. , Dirnagl, U. , Chalmers, I. , Ioannidis, J. P. , … Glasziou, P. (2014). Biomedical research: Increasing value, reducing waste. The Lancet, 383(9912), 101–104. 10.1016/s0140-6736(13)62329-6 24411643

[cch12904-bib-0020] McDonagh, J. E. , & Bateman, B. (2011). ‘Nothing about us without us’: Considerations for research involving young people. Archives of Disease in Childhood – Education and Practice, 97(2), 55–60. 10.1136/adc.2010.197947 21803922

[cch12904-bib-0021] Nap‐van der Vlist, M. N. , Kars, M. C. , van der Sprenkel, E. E. B. , Nijhof, L. N. , Grootenhuis, M. A. , van Geelen, S. M. , van der Ent, C. K. , Swart, J. F. , van Royen‐Kerkhof, A. , van Grotel, M. , van de Putte, E. M. , & Nijhof, S. L. (2019). Daily life participation in childhood chronic disease: A qualitative study. Archives of Disease in Childhood, 105(5), 463–469. 10.1136/archdischild-2019-318062 31748222

[cch12904-bib-0022] Odgers, H. L. , Tong, A. , Lopez‐Vargas, P. , Davidson, A. , Jaffe, A. , McKenzie, A. , Pinkerton, R. , Wake, M. , Richmond, P. , Crowe, S. , Caldwell, P. H. Y. , Hill, S. , Couper, J. , Haddad, S. , Kassai, B. , & Craig, J. C. (2018). Research priority setting in childhood chronic disease: A systematic review. Archives of Disease in Childhood, 103(10), 942–951. 10.1136/archdischild-2017-314631 29643102

[cch12904-bib-0023] Parsons, S. , Thomson, W. , Cresswell, K. , Starling, B. , & McDonagh, J. E. (2017). What do young people with rheumatic disease believe to be important to research about their condition? A UK‐wide study. Pediatric Rheumatology, 15(1), 53. 10.1186/s12969-017-0181-1 28673355 PMC5496376

[cch12904-bib-0024] Sarti, A. , Schalkers, I. , Bunders, J. F. , & Dedding, C. (2017). Around the table with policymakers: Giving voice to children in contexts of poverty and deprivation. Action Research, 16(4), 396–413. 10.1177/1476750317695412

[cch12904-bib-0025] Schalkers, I. , Dedding, C. W. , & Bunders, J. F. (2014). ‘[I would like] a place to be alone, other than the toilet’—Children's perspectives on paediatric hospital care in the Netherlands. Health Expectations, 18(6), 2066–2078. 10.1111/hex.12174 24460634 PMC5810735

[cch12904-bib-0026] Schoemaker, C. G. , Armbrust, W. , Swart, J. F. , Vastert, S. J. , van Loosdregt, J. V. , Verwoerd, A. , Whiting, C. , Cowan, K. , Olsder, W. , Versluis, E. , van Vliet, R. , Fernhout, M. J. , Bookelman, S. L. , Cappon, J. , van den Berg, J. M. , Schatorjé, E. , Muller, P. C. E. H. , Kamphuis, S. , de Boer, J. , … Wulffraat, N. M. (2018). Dutch juvenile idiopathic arthritis patients, carers and clinicians create a research agenda together following the James Lind Alliance method: A study protocol. Pediatric Rheumatology, 16(1), 57. 10.1186/s12969-018-0276-3 30219072 PMC6139167

[cch12904-bib-0027] Schölvinck, A. M. , Pittens, C. A. C. M. , & Broerse, J. E. W. (2020). Patient involvement in agenda‐setting processes in health research policy: A boundary work perspective. Science and Public Policy, 47(2), 246–255.

[cch12904-bib-0028] Thompson, J. , Bissell, P. , Cooper, C. L. , Armitage, C. J. , & Barber, R. (2014). Exploring the impact of patient and public involvement in a cancer research setting. Qualitative Health Research, 24(1), 46–54. 10.1177/1049732313514482 24277776 PMC4509885

[cch12904-bib-0029] Tong, A. , Jones, J. , Craig, J. C. , & Singh‐Grewal, D. (2012). Children's experiences of living with juvenile idiopathic arthritis: A thematic synthesis of qualitative studies. Arthritis Care & Research, 64(9), 1392–1404. 10.1002/acr.21695 22504867

[cch12904-bib-0030] UN General Assembly , Convention on the rights of the child, 20 November United Nations, Treaty Series 1989; vol. 1577, p. 3

[cch12904-bib-0031] van Bijleveld, G. G. , Vetten, M. D. , & Dedding, C. W. (2020). Co‐creating participation tools with children within child protection services: What lessons we can learn from the children. Action Research, 1–17. 10.1177/1476750319899715

[cch12904-bib-0032] van Gulik, E. C. , Verkuil, F. , Barendregt, A. M. , Schonenberg‐Meinema, D. , Rashid, A. N. , Kuijpers, T. W. , van den Berg, J. M. , & Hoving, J. L. (2020). Experiences, perspectives and expectations of adolescents with juvenile idiopathic arthritis regarding future work participation; a qualitative study. Pediatric Rheumatology, 18(1), 33. 10.1186/s12969-020-00429-6 32293467 PMC7158382

[cch12904-bib-0033] Vella‐Baldacchino, M. , Perry, D. C. , Roposch, A. , Nicolaou, N. , Cooke, S. , Ellis, P. , & Theologis, T. (2019). Research priorities in children requiring elective surgery for conditions affecting the lower limbs: A James Lind Alliance Priority Setting Partnership. BMJ Open, 9(12), e033233. 10.1136/bmjopen-2019-033233 PMC6955494 31892663

[cch12904-bib-0034] Verwoerd, A. , Armbrust, W. , Cowan, K. , van den Berg, L. V. , de Boer, J. D. , Bookelman, S. , Britstra, M. , Cappon, J. , Certan, M. , Dedding, C. , van den Haspel, K. , Muller, P. H. , Jongsma, K. , Lelieveld, O. , van Loosdregt, J. , Olsder, W. , Rocha, J. , Schatorjé, E. , Schouten, N. , … Schoemaker, C. G. (2021). Dutch patients, caregivers and healthcare professionals generate first nationwide research agenda for juvenile idiopathic arthritis. Pediatric Rheumatology, 19, 52. 10.1186/s12969-021-00540-2 33827608 PMC8028801

